# Deep Learning for the Automatic Quantification of Pleural Plaques in Asbestos-Exposed Subjects

**DOI:** 10.3390/ijerph19031417

**Published:** 2022-01-27

**Authors:** Ilyes Benlala, Baudouin Denis De Senneville, Gael Dournes, Morgane Menant, Celine Gramond, Isabelle Thaon, Bénédicte Clin, Patrick Brochard, Antoine Gislard, Pascal Andujar, Soizick Chammings, Justine Gallet, Aude Lacourt, Fleur Delva, Christophe Paris, Gilbert Ferretti, Jean-Claude Pairon, François Laurent

**Affiliations:** 1 Faculté de Médecine, Université de Bordeaux, 33000 Bordeaux, France; gael.dournes@u-bordeaux.fr (G.D.); patrick.brochard@chu-bordeaux.fr (P.B.); francois.laurent@chu-bordeaux.fr (F.L.); 2 Service d’Imagerie Médicale Radiologie Diagnostique et Thérapeutique, CHU de Bordeaux, 33000 Bordeaux, France; 3 Centre de Recherche Cardio-Thoracique de Bordeaux, INSERM U1045, Université de Bordeaux, 33000 Bordeaux, France; 4 Mathematical Institute of Bordeaux (IMB), CNRS, INRIA, Bordeaux INP, UMR 5251, Université de Bordeaux, 33400 Talence, France; baudouin.denis-de-senneville@u-bordeaux.fr; 5 Epicene Team, Bordeaux Population Health Research Center, INSERM UMR 1219, Université de Bordeaux, 33000 Bordeaux, France; morgane.menant@u-bordeaux.fr (M.M.); celine.gramond@u-bordeaux.fr (C.G.); justine.gallet@u-bordeaux.fr (J.G.); aude.lacourt@inserm.fr (A.L.); fleur.delva@chu-bordeaux.fr (F.D.); 6 Centre de Consultation de Pathologies Professionnelles, CHRU de Nancy, Université de Lorraine, 54000 Nancy, France; i.thaon@chru-nancy.fr; 7 Service de Santé au Travail et Pathologie Professionnelle, CHU Caen, 14000 Caen, France; clin-b@chu-caen.fr; 8 Faculté de Médecine, Université de Caen, 14000 Caen, France; 9 Service de Médecine du Travail et de Pathologies Professionnelles, CHU de Bordeaux, 33000 Bordeaux, France; 10 Faculté de Médecine, Normandie Université, UNIROUEN, UNICAEN, ABTE, 76000 Rouen, France; Antoine.Gislard@chu-rouen.fr; 11 Centre de Consultations de Pathologie Professionnelle, CHU de Rouen, CEDEX, 76031 Rouen, France; 12 Equipe GEIC20, INSERM U955, 94000 Créteil, France; pascal.andujar@inserm.fr (P.A.); jc.pairon@chicreteil.fr (J.-C.P.); 13 Faculté de Santé, Université Paris-Est Créteil, 94000 Créteil, France; 14 Service de Pathologies Professionnelles et de l’Environnement, Centre Hospitalier Intercommunal Créteil, Institut Santé-Travail Paris-Est, 94000 Créteil, France; 15 Institut Interuniversitaire de Médecine du Travail de Paris-Ile de France, 94000 Créteil, France; soizick.chammings@iimtpif.fr; 16 Service de Santé au Travail et Pathologie Professionnelle, CHU Rennes, 35000 Rennes, France; christophe.paris@inserm.fr; 17 Institut de Recherche en Santé, Environnement et Travail, INSERM U1085, 35000 Rennes, France; 18 INSERM U 1209 IAB, 38700 La Tronche, France; gferretti@chu-grenoble.fr; 19 Domaine de la Merci, Faculté de Médecine, Université Grenoble Alpes, 38706 La Tronche, France; 20 Service de Radiologie Diagnostique et Interventionnelle Nord, CHU Grenoble Alpes, CS 10217, 38043 Grenoble, France

**Keywords:** artificial intelligence, pleural plaques, asbestos exposure

## Abstract

Objective: This study aimed to develop and validate an automated artificial intelligence (AI)-driven quantification of pleural plaques in a population of retired workers previously occupationally exposed to asbestos. Methods: CT scans of former workers previously occupationally exposed to asbestos who participated in the multicenter APEXS (Asbestos PostExposure Survey) study were collected retrospectively between 2010 and 2017 during the second and the third rounds of the survey. A hundred and forty-one participants with pleural plaques identified by expert radiologists at the 2nd and the 3rd CT screenings were included. Maximum Intensity Projection (MIP) with 5 mm thickness was used to reduce the number of CT slices for manual delineation. A Deep Learning AI algorithm using 2D-convolutional neural networks was trained with 8280 images from 138 CT scans of 69 participants for the semantic labeling of Pleural Plaques (PP). In all, 2160 CT images from 36 CT scans of 18 participants were used for AI testing versus ground-truth labels (GT). The clinical validity of the method was evaluated longitudinally in 54 participants with pleural plaques. Results: The concordance correlation coefficient (CCC) between AI-driven and GT was almost perfect (>0.98) for the volume extent of both PP and calcified PP. The 2D pixel similarity overlap of AI versus GT was good (DICE = 0.63) for PP, whether they were calcified or not, and very good (DICE = 0.82) for calcified PP. A longitudinal comparison of the volumetric extent of PP showed a significant increase in PP volumes (*p* < 0.001) between the 2nd and the 3rd CT screenings with an average delay of 5 years. Conclusions: AI allows a fully automated volumetric quantification of pleural plaques showing volumetric progression of PP over a five-year period. The reproducible PP volume evaluation may enable further investigations for the comprehension of the unclear relationships between pleural plaques and both respiratory function and occurrence of thoracic malignancy.

## 1. Introduction

The association between occupational asbestos exposure and the increase in benign and malignant respiratory diseases has been demonstrated through several studies in the last decades [1]. Among these asbestos exposure manifestations, pleural plaques (PP) are the most frequent effects of such exposure [2,3,4]. Chest x-rays are widely used in the screening for respiratory diseases in the context of occupational asbestos exposure. However, computed tomography (CT) scans showed higher sensitivity and specificity for both pleural and parenchymal abnormalities [5]. Pleural plaques are localized, well-demarcated areas of thickening in the parietal pleura consisting of hyaline fibrosis [6,7]. They may have a smooth or irregular/nodular surface with the parenchyma, and they exhibit a soft-tissue attenuation with sometimes calcifications [1]. In France, workers are entitled to financial compensation and early retirement in the case of asbestos-related disease, including pleural plaques. Indeed, pleural plaques are considered as the hallmark of asbestos exposure, and their prevalence is correlated to several determinants of exposure [8]. Despite several studies, there is still some controversy about the issue of whether or not pleural plaques are responsible for respiratory function impairment [9,10,11]. Furthermore, studies [12,13] have suggested that PP might be an independent risk factor for malignancy in workers previously occupationally exposed to asbestos. However, the progression of PP extent has not been studied yet due to the lack of consensual quantitative methods. Few studies focused on quantifying PP extent either visually [9,14] or using manual/semi-automated methods [15,16,17]. These methods demonstrate fair to good reproducibility, but they are time-consuming and cumbersome.

In the last few years, Deep learning (DL) has been considered as the most reliable technique for medical image segmentation [18]. However, to the best of our knowledge, pleural plaques segmentation using DL has never been reported. Thus, using AI-driven PP segmentation may allow reproducible quantification of pleural plaques. This may help to further investigate relationships between asbestos exposure and benign and malignant respiratory diseases. Therefore, the objective of this study was to validate a novel fully automated quantitative method for PP evaluation based on DL.

## 2. Material and Methods

### 2.1. Study Design

This is a retrospective cohort study involving retired workers exposed to asbestos during their working life and without already compensated asbestos-related disease. The hospital ethics committee approved the study (CPPRB Paris-Cochin n°1946 (2002), CPP Ile De France III n°1946/11/02-02 (2010)). All participants received information about the study and gave their written informed consent.

### 2.2. Settings

Asbestos-Related Diseases COhort (ARDCO) is a French surveillance program where retired workers exposed to asbestos during their working life were included between October 2003 and December 2005 in four regions. They benefited from a free medical check-up, including chest CT scan and pulmonary function tests repeated every 5 years. Chest CT scans were sent to the regional coordinating centers and constituted the Asbestos Post EXposure Survey (APEXS) study.

### 2.3. Participants

**Figure 1:**
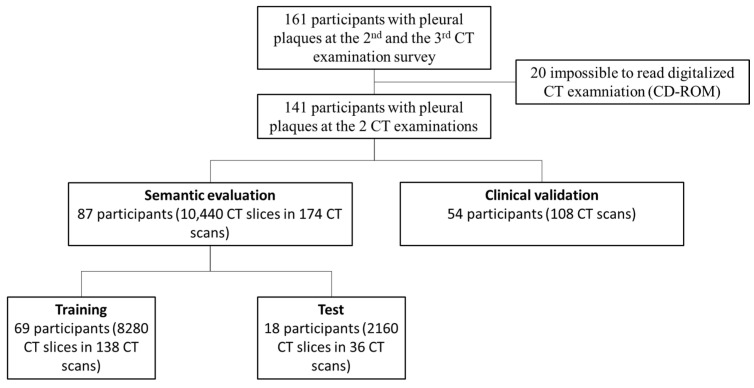
Study Flow-Chart of selected patients with related Computed Tomography (CT). CT = computed tomography.

The study analyses the second and third round of screening (collected in 2010 and 2017 respectively) since CT scans of the first round were of insufficient quality (5 mm slice thickness without high resolution CT acquisition and/ or the digital format was missing). Finally, participants with pleural plaques identified by expert radiologists at the 2nd and the 3rd CT screenings were included (Figure 1).

### 2.4. Data

We extracted data from the APEXS survey, managed by Institut Interuniversitaire de Médecine du Travail de Paris-Ile de France, Créteil, France. Data contain patients’ CT scans and characteristics (age: years; gender: Male/Female; smoking status: Never smoker/Ex-smoker/Current smoker; Asbestos exposure: Duration (years) and Time since first exposure (years); Related conditions on CT scans: Lung nodule/Asbestosis/Lung cancer).

### 2.5. Chest CT Scans Visual Analysis

To establish the diagnosis of pleural plaques, a consensus of three expert radiologists was used as previously reported [14]. Pleural plaques were defined according to the Fleischner society glossary of terms [19]. The thickness of PP was evaluated by classifying the most thickened plaque into four categories: <2 mm, 2–5 mm, 5–10 mm, and >10 mm. The extent of pleural plaques was classified into four categories according to the cumulative area of pleural plaques detected on each section and virtually reported on the single section at the level of the carina. The following thresholds were used: <1 cm length, between 1 cm and one-quarter of the perimeter of the hemithorax, between one-quarter and one-half of the perimeter of the hemithorax, more than one-half of the perimeter of the hemithorax. The number of PP was also reported according to three categories: equal to one; equal to two and more than two. The product of (the thickness x the cumulative area x number of plaques) constituted the visual extent score of PP. The standardized readings were randomized, blinded to other experts’ readings.

### 2.6. AI Training Framework

Briefly, trained thoracic radiologists blinded to the participants’ characteristics have manually delineated pleural plaques on standard kernel reconstructed CT images in a random fashion. The manual segmentation was considered as the ground truth (GT). A pre-processing using thin maximum intensity projection (MIP) was used to limit the number of images for the manual segmentation. A five-millimeter slice thickness for MIP was used. Therefore, the number of chest CT slices decreased from 300 to 60 approximately.

A 2D-convolutional neural network (CNN) was trained utilizing the Training cohort. We used one single input channel (i.e., the CT image). We used the U-Net architecture presented in Figure S1 [20] which shown good performance for the segmentation of small regions [21,22]. To reduce memory consumption without impacting performance, we selected a basis of 24 filters of 3 × 3 − 24 for the first layer, 48 for the second and so on, as proposed in [23]. The loss function (L) was a combination of binary cross-entropy ($L_{BCE}$) [24] and Dice loss ($L_{DL}$) [25] which is demonstrated to be well suited for imbalanced structure segmentation [26]. L was defined as:

$$L=L_{BCE}+L_{DL}\;L_{BCE}=-\left(y\;\log\left(\hat{y}\right)+\left(1-\;y\right)\log\left(1-\hat{y}\right)\right)\;L_{DL}=1-\frac{2y\hat{y}+1}{y+\hat{y}+1}$$

y and $\hat{y}$ being the true and the predicted value of the CNN, respectively.

The following parameters were employed: input resolution = 512 × 512, batch size = 1, optimizer = Adam [27], learning rate = 0.001, epoch = 200, dropout = 0.5 after each block of the descending path, upsampling based on trilinear interpolation in the decoder, skip connections between encoder and decoder based on concatenation. To improve the ability for CNN to generalize, the training dataset was expanded through data augmentation (horizontal/vertical flips were applied during training). Our test platform was an Intel Xeon E5-2683 2.4 GHz equipped by a GPU Nvidia Tesla P100 with 16 GB of memory. Our implementation was done using Tensorflow 1.4 and Keras 2.2.4.

### 2.7. Test Cohort

2D-similarity and 3D-concordance assessments between AI-driven and GT test labels were performed. Calcified pleural plaques were identified by using a threshold of 100 Hounsfield units (HU) on the masks of PP, and their similarity and volume concordance was also assessed. A longitudinal comparison of PP volume progression was evaluated. Correlation with the visual extent score of PP was assessed.

### 2.8. Clinical Validation Cohort

Longitudinal paired-comparison analyses of PP volumes were performed. AI-driven PP volume quantification was performed on both native CT slices (slice thickness 1–1.25 mm), and MIP processed CT images. In addition, correlation with PP visual extent score was evaluated.

### 2.9. Reproducibility Assessment

AI-driven measurements were performed twice in the test cohort, while two observers blinded to any other data and the other observer labels, manually segmented a random subset of 20 CT exams. For intra-observer reproducibility, the subset of 20 CTs was re-segmented manually by the first observer a month later to avoid recall bias.

### 2.10. Statistical Analysis

Data were expressed as medians with a 95% confidence interval (CI). The similarity was assessed by calculating the balanced accuracy, Sorensen–DICE similarity coefficient (DICE), precision, and recall [28]. Concordance was assessed by concordance correlation coefficient (CCC) and Bland–Altman analysis [29]. Normality was evaluated using Shapiro–Wilk test. Spearman’s rho coefficient assessed correlations. A comparison of paired-medians was made by the Wilcoxon-rank test. Correlation coefficients were classified as null (=0) to almost perfect (≥0.95) [30].

## 3. Results

### 3.1. Participants

The study population consisted of 141 asbestos-exposed participants with pleural plaques at both the second and the third screening rounds after excluding 20 participants with unreadable CD-ROMs (Figure 1). Eighty-seven participants constituted the semantic evaluation cohort. Stratified randomization based on the CT scanner models was used to split the original CT dataset into two non-overlapping groups [31], i.e., Train cohort (*n* = 8280 CT slices from 138 CT examinations of 69 participants) and independent Test cohort (*n* = 2160 CT slices from 36 CT examinations of 18 participants) without cross-validation. There were 13 CT models from four major manufacturers (Supplementary Table S1).

Finally, to independently evaluate the clinical relevance and validity of the AI-driven PP quantification, a clinical validation cohort was constituted (*n* = 54 participants).

Age, smoking status, asbestos exposure characteristics and related lung conditions are summarized in Table 1.

### 3.2. Similarity and Concordance of Test Cohort

**Figure 2:**
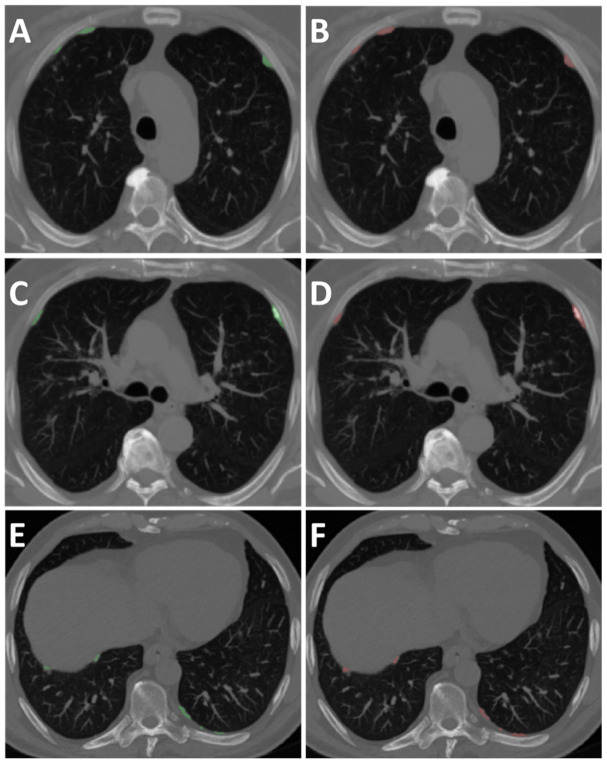
Axial MIP (5 mm) CT images of 80-year-old male. Left panel represents GT pleural plaques segmentation (green). Right panel represents AI pleural plaques segmentation (red). Note the different pleural plaques localization at three levels of the chest. Anterolateral PPs at the aortic arch level (**A**,**B**); Calcified PPs at the carina level (**C**,**D**) Posterolateral and diaphragmatic PPs at the lower chest (**E**,**F**). Legends: MIP = maximum intensity projection; CT = computed tomography; GT= ground truth; AI = artificial intelligence.

Axial CT slices of the 36 CT examinations (*n* = 2160 slices) were shuffled randomly before being segmented by the 2D-CNN. The 2D pixel similarity overlap of AI versus GT was good (DICE = 0.63). The similarity overlap for calcified pleural plaques was found very good (DICE = 0.82) (Figure 2, Table 2). The AI-driven PP volume segmentation’s balanced accuracy for both PP and calcified PP was good to very good (0.78 and 0.90, respectively). (Table 2).

After re-assigning CT slices to their initial examinations (*n* = 36 CTs), the 3D concordance between AI-driven volume calculations and GT labels was almost perfect for both PP and calcified PP (CCC > 0.98 95%CI: 0.96–099) (Table 2). At the Bland–Altman analysis, the mean difference between AI-driven volume and GT volume was small (less than 2.3 mL for PP and calcified PP) (Table 2).

### 3.3. Correlations with Visual Pleural Plaques Extent Score

There was a significant moderate correlation between the AI-driven PP volumetric quantification and the PP visual extent score in the test cohort (rho = 0.66, *p* < 0.001). The correlation coefficient was similar to that of the GT label (rho = 0.68, *p* < 0.001). Similarly, in the clinical validation cohort, there was a significant correlation between the AI-driven PP volume and the visual PP extent (rho = 0.56, *p* < 0.001).

### 3.4. Longitudinal Comparison of Pleural Plaques Volume Progression

**Figure 3:**
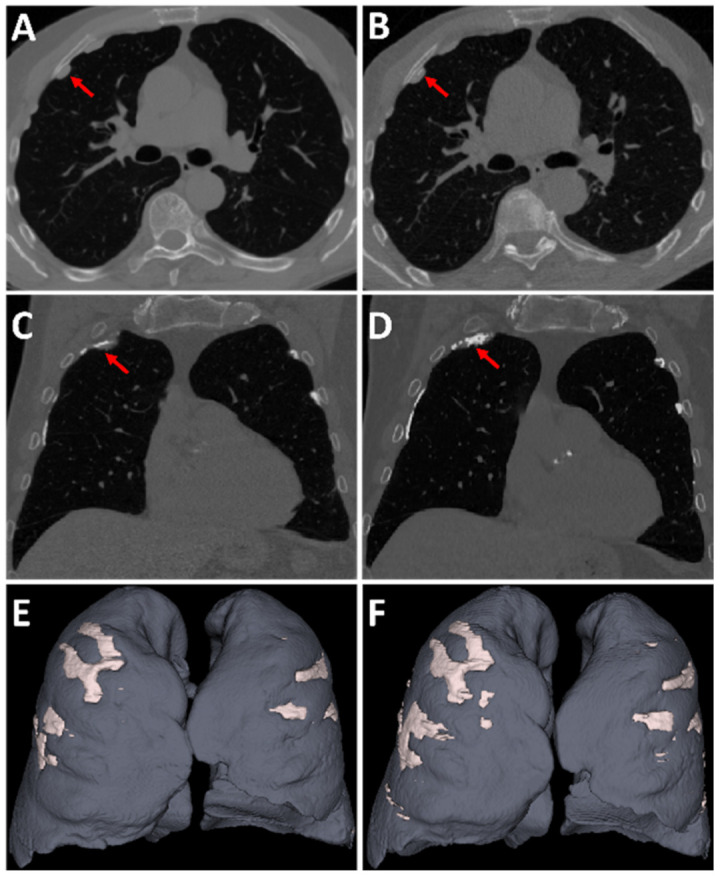
CT images of 71 years-old male at the 2nd (left panel) and the 3rd (right panel) CT screening rounds. Note the increase in pleural plaques volume ((**E**) 28.01 mL and (**F**) 49.25 mL), with the increase in calcifications (red arrows). (**A**,**B**) Axial native CT images (1 mm slice thickness); (**C**,**D**) Coronal native CT images; (**E**,**F**) 3D-volume rendering of CT images.

In the clinical validation cohort, participants had a significant increase in AI-driven volumetric quantification of both PP and calcified PP between the second and the third rounds of CT screening (*p* < 0.001) (Figure 3, Table 3). The median differences were 5.71 mL (95%CI: 3.57–9.48) and 2.85 mL (95%CI: 1.62–7.76) for PP and calcified PP, respectively. The median percentage of the increase was 70% (95%CI: 38–123%) and 143% (95%CI: 85–230%) for PP and calcified PP, respectively. Similarly, in the test cohort, there was an increase in both AI-driven PP volume and GT PP volume between the two rounds of CT screening (*p* < 0.001). (Supplementary Table S2). There was also an increase in the visual extent score of PP between the two CT scans for both the test cohort and the clinical validation cohort (*p* = 0.003) (Supplementary Table S3).

Moreover, there was no significant difference between AI-driven PP volume quantification using native CT images and thin MIP pre-processed CT images (Supplementary Table S4).

### 3.5. Reproducibility of Evaluation

As expected, the quantitative measurements of AI-driven segmentation had an almost perfect reproducibility when repeated twice in all 36 CT scans (2160 axial CT slices) of the Test cohort (DICE > 0.99) (Table 4). The similarity between manual segmentations performed by either the same observer or two independent observers was also assessed in a subset of 20 CTs (1200 axial CT slices). This additional evaluation showed a DICE coefficient of 0.72 and 0.75 for PP and calcified PP segmentations, respectively, in the inter-observer evaluation; and 0.87 and 0.89 in the intra-observer evaluation. The concordance correlation coefficients for the manual and AI-driven PP quantification were excellent (>0.98). (Table 4). In addition, the time requested to perform a manual GT was higher (i.e., a median time of 15 min) than that requested to perform AI-driven labeling (i.e., a median time of 18 s for thin MIP images and 90 s for native images).

## 4. Discussion

This study shows that AI-driven quantitative measurement of pleural plaques is a reliable, fully automatic technique that can provide relevant information about pleural plaques progression in a population of retired workers previously occupationally exposed to asbestos. The method showed good similarity and very good concordance with manual segmentation of pleural plaques. Volumetric measurements of pleural plaques demonstrated a significant increase between two rounds of CT screening with an average delay of five years. In addition, the PP volume quantification showed a significant correlation with expert visual CT score of PP extent.

This study has some limitations. First, this was a retrospective first attempt to evaluate the novel method. Large prospective studies may bring more insights into pleural plaques volume quantification and asbestos exposure. Second, detection of pleural plaques was out of scope since the diagnosis of pleural plaque presence is easily made by an expert radiologist in few seconds. However, using the same dataset, AI algorithms could be trained for detection tasks. Further evaluation using alternative strategies such as multiple CNNs with major voting [31], multiplane consensus labeling [32], or 3D AI algorithms [33] would be worth evaluating, albeit with a heavy computational burden. We used 2D-CNN to train the model in a slice-by-slice fashion to account for pleural plaque localization, or shape heterogeneity. Moreover, using CT slices as inputs, the Training dataset was large enough (8280 CT slices) for accurate development of the model [34]. Using 3D-CNN would limit the Training dataset in addition to the anisotropy due to the MIP pre-processing that may impede the generalizability of the model. Indeed, we demonstrated that by using 2D-CNN, PP segmentation was similar whether performed on native CT images or pre-processed 5 mm thin MIP images.

To the best of our knowledge, our study is the first that aimed to develop and validate AI-driven quantification of pleural plaques in a population of retired workers previously occupationally exposed to asbestos. Indeed, Sousa et al. [35] have reported a combination of CNN and support vector machines (SVM) classification based on 3D patch selection to detect pleural plaques without segmentation or quantitative measurement. They showed good to a very good Kappa for classifying 3D patches into pleural plaque or healthy tissue. However, their dataset was too small and heavily unbalanced, with 22% of CTs with pleural plaques (16/71), which may have led to overfitting.

Manual segmentation of pleural plaques could be a tedious and time-consuming task, which may have prevented the radiologists’ community from tackling the issue of automatic quantification using DL. In this study, to facilitate manual segmentation, CT scans were pre-processed using maximum intensity projection. Therefore, the number of chest CT slices decreased from 300 to 60 approximatively. The rationale of using a thin MIP over a maximum of five slices (i.e., 5 mm of thickness) is that pleural plaques show attenuation values higher than the surrounding structures (i.e., lung, fat), which facilitate the detection and delineation of PP. On the contrary, the ribs, which are often in contact with PP, show a higher attenuation value. However, a MIP along five-millimeter of thickness would prevent the overlap of PP and costal structures. Moreover, with a thin MIP, partial volume artifacts at the pleura thoracic wall interfaces were limited, especially in non-vertical areas such as the diaphragm and the apices. In addition, we demonstrated that AI-driven pleural plaques volume derived from either native CT images or MIP pre-processed CT images was not different. In the last few years, we have evaluated semi-automated segmentation of pleural plaques [17] and showed very good reproducibility of the PP measurements. Nonetheless, since the semi-automated technique was based on a convex-hull algorithm, many false positives and false negatives occurred, and a substantial amount of manual editing was required. On the other hand, visual scoring of pleural plaques was extensively used in asbestos exposure studies showing good kappa coefficients for binary evaluations but with poor or moderate kappa coefficients for thickness or extent pleural plaques evaluations [14].

The use of DL in medical imaging in the last few years, allowed a giant leap of image segmentation with less and less of manual editing [18]. Indeed, with fine-tuning and the constant increase in medical imaging databases, CNNs will improve dramatically, leading to fully automated tasks performed by the machine.

In our study, an independent Testing cohort of 2160 CT slices was used, including the whole chest without preselecting a few slices with pleural plaques. Thus, this provided an extensive overview of the performance of the technique. We reported a DICE coefficient of 0.63 for PP segmentation, which represents a good overlap index, knowing that pleural plaques may have less than one millimeter of thickness and a very limited extent [36]. Indeed, the Dice coefficient is known to assign a lower score for very small structures involving few pixels with insufficient overlap [28]. Thus, any misclassified pixel would have decreased substantially the Dice similarity coefficient. Regarding calcified PP, the Dice coefficient was found very good (0.82), which may be related to relatively easy classification of the calcified pixels by the model due to their high attenuation value. Since the Dice metric does not include true negatives, CCC was used to assess PP volumes at the participant level showing excellent 3D concordance with GT. Furthermore, we showed that the AI-driven volumetric PP quantification was consistent with the visual extent score of PP.

This is the first time that longitudinal evaluation of pleural plaques has been reported. We showed a progression of pleural plaques volume through the five-year delays between the two rounds of CT screening. This was expected since pleural plaques were demonstrated to be associated with asbestos exposure latency [8]. Nevertheless, our volumetric quantification objectively depicts the progression of pleural plaques. In addition, we reported an increase in calcified pleural plaques between the two rounds of CT screening. Indeed, calcified pleural plaques are also known to be related to latency [37].

Relationships between the prevalence of pleural plaques and benign or malignant respiratory diseases were extensively evaluated in the scientific literature [38]. The presence of pleural plaques was found to be an independent risk factor for lung cancer or mesothelioma [12,13]. In addition, the extent of pleural plaques evaluated using visual score showed a correlation with the decline of forced vital capacity [9]. Moreover, correlation between pleural plaques extent using visual scores and asbestos exposure duration and latency has been already reported [37]. In our study, we showed significant correlations between AI-driven PP volume quantification and visual scoring. Hence, this reproducible novel method offers promising multiple perspectives for the researchers in the field of respiratory diseases related to asbestos exposure.

The AI-driven technique has several advantages in comparison to visual or semi-automated evaluations of pleural plaques extent. A few seconds are needed to accomplish the whole segmentation of pleural plaques with an almost perfect reproducibility. In contrast, the visual/manual scores are time-consuming with moderate reproducibility. This detailed quantification of pleural and calcified pleural plaques may be more relevant in evaluating the relationships with benign and malignant respiratory diseases.

Although four manufacturers were represented in this study, additional evaluation with other manufacturers and CT models would be desirable to increase the generalizability of the AI model.

## 5. Conclusions

We showed for the first time that an automated AI-driven quantification of pleural plaques is feasible, which may help researchers to decipher the unclear relationships between pleural plaques and both respiratory function and chest malignancy.

## Figures and Tables

**Table 1:** Patients’ characteristics of the 141 asbestos-exposed retired workers from the French Asbestos Related Diseases Cohort followed between 2010 and 2017 (all had pleural plaques on CT scan at both the second and third screening rounds).

		Training Cohort	Test Cohort	Clinical Validation Cohort
		(*n* = 69)	(*n* = 18)	(*n* = 54)
**Age**	Years	71 ± 4	71 ± 5	70 ± 4
**Gender**	Male/Female	68/1	18/0	54/0
**Smoking status**	Never smoker	14	5	18
	Ex smoker	51	11	32
	Current Smoker	4	2	4
**Asbestos exposure**				
	Total duration (y)	36 (34–38)	38 (35–39)	35 (33–37)
	Time since first exposure (y)	52 ± 5	53 ± 6	52 ± 4
**Related conditions**				
	Lung nodule (yes/no)	15/54	9/9	29/25
	Asbestosis (yes/no)	4/65	1/17	3/51
	Lung cancer (yes/no)	3/66	0/18	4/50

Data are means ± sd or medians (95% CI) for continuous variables and absolute value for categorical variables.

**Table 2:** 2D pixel similarity and 3D volume concordance between AI-driven and Ground Truth in the Test cohort (18 patients).

**2D Pixel Similarity**	**Pleural Plaques**	**Calcified Pleural Plaques**
*N* = 2160 axial CT slices		
**Balanced Accuracy**	0.78	0.90
**DICE**	0.63	0.82
**Recall**	0.56	0.80
**Precision**	0.71	0.84
**3D Volume Extent (mL)**	**Pleural Plaques**	**Calcified Pleural Plaques**
*N* = 36 CT scans		
**Concordance:** CCC (95% CI)	0.98 (0.96; 0.99)	0.99 (0.99; 0.99)
**Bland–Altman (mL):** Mean difference (LOA)	2.3 (−17.4; 22)	−0.3 (−2.2; 1.6)

Legend: AI = artificial intelligence; CCC = concordance correlation coefficient; CI = confidence interval; LOA = limits of agreement.

**Table 3:** Longitudinal comparison of AI-driven pleural plaques quantification in the clinical validation cohort (*n* = 54 patients).

		CT^2nd^	CT^3rd^	*p*-Value
**AI-driven quantification**				
**Pleural Plaques (mL)**	Median	7.1	12.1	<0.001
	95%CI	(4.4–11.5)	(9.8–16.9)	
**Calcified Pleural Plaques (mL)**				
	Median	1.3	3.5	<0.001
	95%CI	(0.6–2.6)	(2.2–5.3)	

Legend: AI = artificial intelligence; CI = confidence interval; CTx = computed tomography at the 2nd or the 3rd screening round.

**Table 4:** Reproducibility of AI and manual Pleural Plaques quantification.

	Pleural Plaques		Calcified Pleural Plaques	
**Comparisons**	2D ^1^	3D ^2^	2D ^1^	3D ^2^
**AI vs. AI** (*n* = 2160 CT slices in 36 CT)	>0.99	>0.99 [0.99–1]	>0.99	>0.99 [0.99–1]
**Manual_1_ vs. Manual_2_** (*n* = 1200 CT slices in 20 CT)	0.72	0.98 [0.95–0.99]	0.75	0.98 [0.95–0.99]
**Manual_1_ vs. Manual_1_** (*n* = 1200 CT slices in 20 CT)	0.87	0.98 [0.97–0.99]	0.89	0.99 [0.97–0.99]

Legends: Manual_1_ = segmentation performed by the first Observer; Manual_2_ = segmentation performed by the second Observer; AI = artificial intelligence; CT=computed tomography. ^1^ 2D pixel similarity (dice); ^2^ 3D volume extent (ml) (CCC).

## Data Availability

The data presented in this study are available on request from the corresponding author. The data are not publicly available due to privacy.

## Supplementary Materials

The following are available online https://www.mdpi.com/article/10.3390/ijerph19031417/s1. Supplementary Table S1: Characteristics of CT scans. Supplementary Table S2: longitudinal comparison of manual and AI-driven pleural plaques quantification in the Test cohort. Supplementary Table S3: longitudinal comparison of Visual evaluation of PP extent. Supplementary Table S4: comparison of pleural plaques volume in the clinical validation cohort using Maximum Intensity projection Vs Native thin slices. Supplementary Figure S1. Illustration of the 2D U-Net architecture used for the segmentation of pleural plaques.
