# Supplementary material for: Deep Learning for the Automatic Quantification of Pleural Plaques in Asbestos-Exposed Subjects
Source: Int J Environ Res Public Health. 2022 Jan 27;19(3):1417. doi: 10.3390/ijerph19031417 (PMC8835296; doi:10.3390/ijerph19031417)
Supplement: Supplementary file 1 [file ijerph-19-01417-s001.zip › ijerph-1535567-supplementary.pdf]

Supplementary Tables

Supplemental Table S1. Characteristics of CT scans					
Groups	Machine Brand	n	kV	mAs	Slice thickness (mm)
Training	GE (LightSpeed, BrightSpeed, Optima, Revolution) ®	81	(100-140)	(10-150)	5 (MIP)
	Siemens Somatom (Sensation, Scope, Definition, Emotion) ®	27			
	Philips (Brillance, Ingenuity, Mx) ®	21			
	Toshiba (Aquilon, Aquilon Prime)®	9			
Test	GE (LightSpeed, BrightSpeed, Optima, Revolution) ®	22	(100-140)	(10-150)	5 (MIP)
	Siemens Somatom (Sensation, Scope, Definition, Emotion) ®	7			
	Philips (Brillance, Ingenuity, Mx) ®	5			
	Toshiba (Aquilon, Aquilon Prime)®	2			
Clinical Validation	GE (LightSpeed, BrightSpeed, Optima, Revolution) ®	75	(100-140)	(10-150)	(1-1.25)
	Siemens Somatom (Sensation, Scope, Definition, Emotion) ®	20			
	Philips (Brillance, Ingenuity, Mx) ®	11			
	Toshiba (Aquilon, Aquilon Prime)®	2			
Legends : kV ; kilovoltage, mAs ; milliampere seconde MIP; maximum intensity projection					

Supplemental Table S2. longitudinal comparison of manual and AI-driven pleural plaques quantification in the Test cohort

Test cohort (n=18)				
		CT^{2nd}	CT^{3rd}	p-value
**Manual quantification**				
**Pleural Plaques (ml)**	Median	11.7	22.1	**<0.001**
	95%CI	(5.4-29.9)	(9.55-45.3)	
**Calcified Pleural Plaques (ml)**				
	Median	0.9	2.1	**0.003**
	95%CI	(0.1-2.9)	(0.7-5.7)	
**AI-driven quantification**				
**Pleural Plaques (ml)**	Median	12.4	18.3	**<0.001**
	95%CI	(4.8-24.4)	(9.2-38.8)	
**Calcified Pleural Plaques (ml)**				
	Median	0.9	2.6	**0.001**
	95%CI	(0.1-3.2)	(0.8-6.8)	

Legend: AI=artificial intelligence; CI=confidence interval; CTx=computed tomography at the 2nd or the 3rd screening round

Supplemental Table S3. longitudinal comparison of Visual evaluation of PP extent

		CT^{2nd}	CT^{3rd}	p-value
**Test Cohort (n=18)**				
**PP visual extent score**	Median	20	30	**0.003**
	95%CI	(20-30)	(20-45)	
**Clinical Validation cohort (n=54)**				
**PP visual extent score**	Median	20	20	**0.003**
	95%CI	(18.6-20)	(20-20)	

Legend: AI=artificial intelligence; CI=confidence interval; CTx=computed tomography at the 2nd or the 3rd screening round

Supplemental Table S4. comparison of pleural plaques volume in the clinical validation cohort using Maximum Intensity projection Vs Native thin slices

**Clinical Validation cohort (n=108)**				
		**MIP**	**Native**	**p-value**
**AI-driven quantification**				
**Pleural Plaques (ml)**	Median	8.2	10.4	**0.12**
	95%CI	(5.8-12.9)	(7.1-12.7)	
**Calcified Pleural Plaques (ml)**				
	Median	1.3	2.5	**0.82**
	95%CI	(0.5-2.3)	(1.5-3.1)	

Legend: AI=artificial intelligence; CI=confidence interval; MIP=maximum intensity projection

Supplementary figure

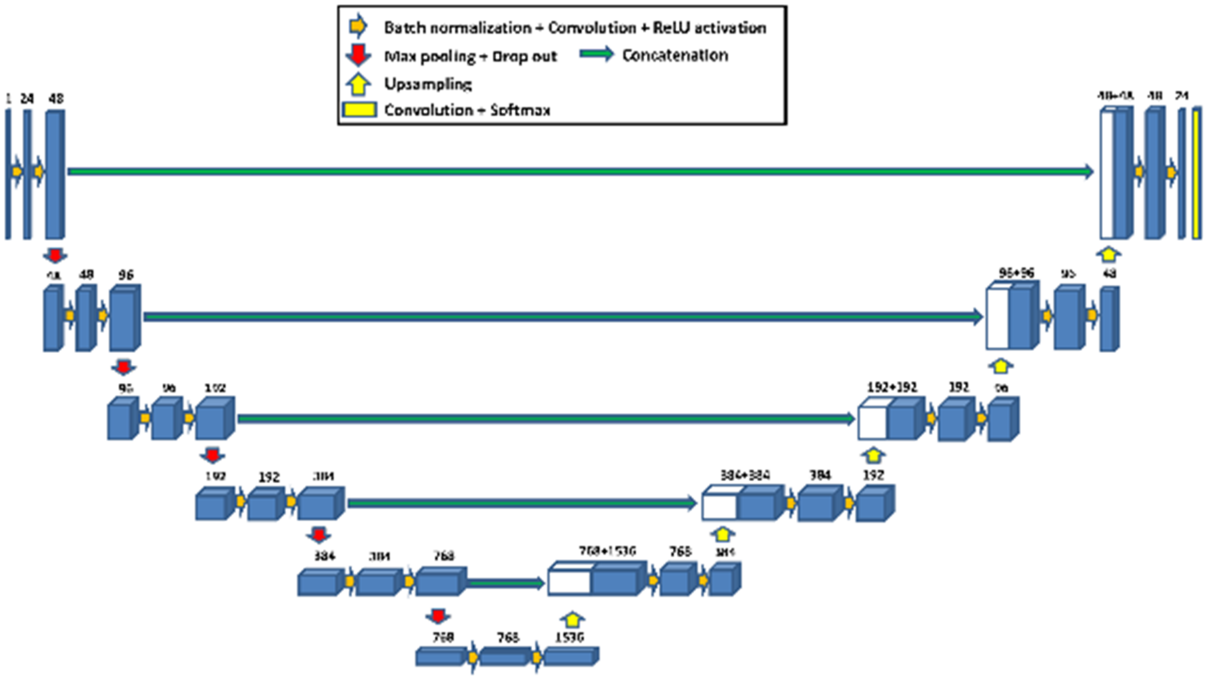

Supplementary Figure S1. Illustration of the 2D U-Net architecture used for the segmentation of pleural plaques. The number of 3×3 filters is indicated on the top of each block.
